# Supplementary material for: Fabrication and Characterization of a Lead-Free Cesium Bismuth Iodide Perovskite through Antisolvent-Assisted Crystallization
Source: Nanomaterials (Basel). 2024 Apr 2;14(7):626. doi: 10.3390/nano14070626 (PMC11013909; doi:10.3390/nano14070626)
Supplement: Supplementary file 1 [file nanomaterials-14-00626-s001.zip › nanomaterials-2920628-supplementary.docx]

| 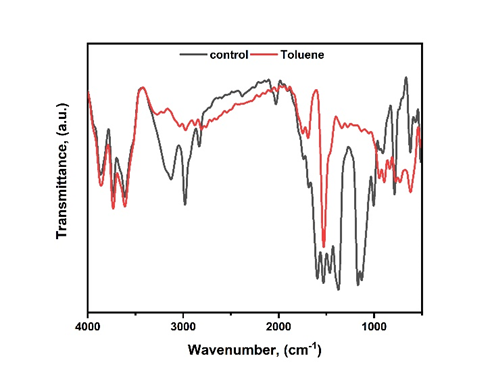 | 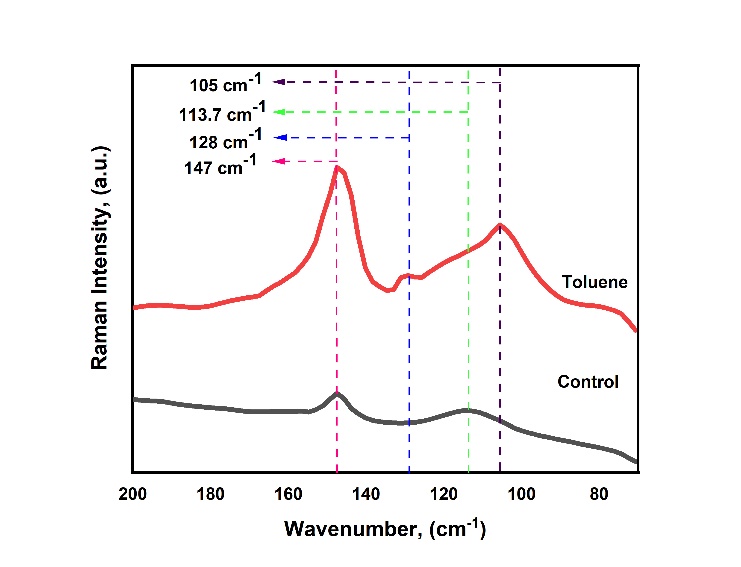 |
| --- | --- |

Figure S1 (a) Ftir at a broder wavenumber from 4000cm-1 and raman spectra for a wavenumber between 70cm-1 and 200cm-1.

| 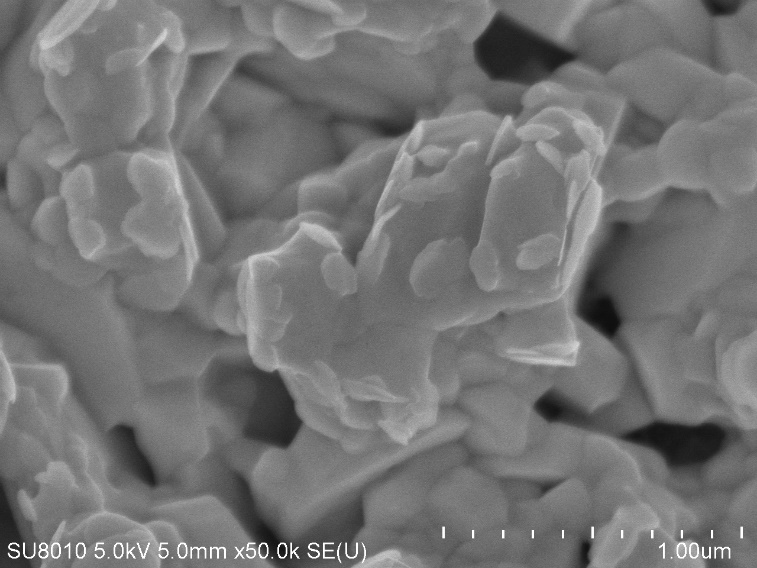  **a** | 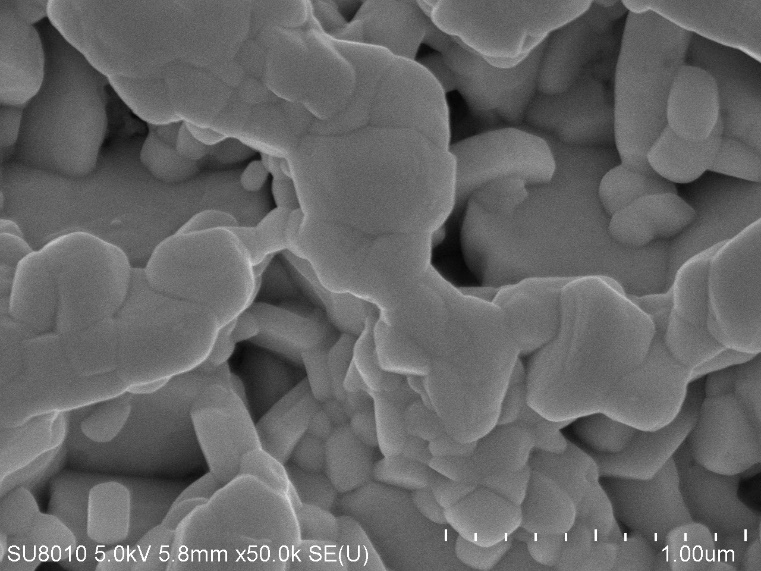  **b** |
| --- | --- |

Figure S2 (a) SEM analyisis for control, (b) for toluene treated film.

Table S1.

|  | A | B | $\boldsymbol{\tau}$**_1_** | $\tau_{2}$ | $\tau_{av}$ |
| --- | --- | --- | --- | --- | --- |
| Control | **610.89** | **8.50** | **3.534E-10** | **4.358E-09** | 0.940129192 |
| Toluene | 575.3121 | 7.03093 | 3.611E-10 | 6.679E-09 | 1.525922815 |
|  |  |  |  |  |  |
